# The sow vaginal and gut microbiota associated with longevity and reproductive performance

**DOI:** 10.1186/s40104-024-01140-2

**Published:** 2025-01-07

**Authors:** Ziyu Liu, Tsungcheng Tsai, Bin Zuo, Samantha Howe, Jason E. Farrar, Christopher E. Randolph, Charles V. Maxwell, Jiangchao Zhao

**Affiliations:** 1 Department of Animal Science, University of Arkansas, Fayetteville, AR USA; 2 Arkansas Children’s Research Institute, Little Rock, AR USA; 3 College of Animal Science, South China Agricultural University, Guangzhou, Guangdong China

**Keywords:** Longevity, Parity, Rectal microbiome, Reproductive performance, Sows, Vaginal microbiome

## Abstract

**Background:**

Sow longevity and reproductivity are essential in the modern swine industry. Although many studies have focused on the genetic and genomic factors for selection, little is known about the associations between the microbiome and sows with longevity in reproduction.

**Results:**

In this study, we collected and sequenced rectal and vaginal swabs from 48 sows, nine of which completed up to four parities (U4P group), exhibiting reproductive longevity. We first identified predictors of sow longevity in the rectum (e.g., *Akkermansia*) and vagina (e.g., *Lactobacillus*) of the U4P group using RandomForest in the early breeding stage of the first parity. Interestingly, these bacteria in the U4P group showed decreased predicted KEGG gene abundance involved in the biosynthesis of amino acids. Then, we tracked the longitudinal changes of the microbiome over four parities in the U4P sows. LEfSe analysis revealed parity-associated bacteria that existed in both the rectum and vagina (e.g., *Streptococcus* in Parity 1, *Lactobacillus* in Parity 2, *Veillonella* in Parity 4). We also identified patterns of bacterial change between the early breeding stage (d 0) and d 110, such as *Streptococcus*, which was decreased in all four parties. Furthermore, sows in the U4P group with longevity potential also showed better reproductive performance. Finally, we discovered bacterial predictors (e.g., *Prevotellaceae NK3B31 group*) for the total number of piglets born throughout the four parities in both the rectum and vagina.

**Conclusions:**

This study highlights how the rectal and vaginal microbiome in sows with longevity in reproduction changes within four parities. The identification of parity-associated, pregnancy-related, and reproductive performance-correlated bacteria provides the foundation for targeted microbiome modulation to improve animal production.

**Supplementary Information:**

The online version contains supplementary material available at 10.1186/s40104-024-01140-2.

## Background

The modern breeding of terminal line swine, selected for enhanced reproductive traits [1] such as litter size, exacerbates the challenge for the primiparous sows to obtain sufficient nutrients during lactation, creating excessive weight loss and bone density reduction, leading to failure to rebreed for subsequent reproductive cycles [2]. These challenges are found in multiparous sows to a lesser extent as parity progresses. Therefore, it is often observed that reproductive performance continuously increases and peaks between parities 3 and 4 [3]. However, sows that fail to rebreed and are subsequently removed from the production cycle can cause significant economic losses in the swine industry.

Parity, which refers to the number of litters a sow has given birth to, is a crucial metric of sow reproductivity in breeding operations [4]. Reproduction traits, including litter size, piglet birth weight, and progeny weight gain during lactation, are substantially affected by parity due to the impact of young sows’ still-developing physiological functions and requirements on their milk yield and milk composition [5]. In addition, parity also affects sow growth, feed efficiency [6], and birth intervals [7]. Recycling of sows through multiple parities and promoting reproductive performance increases the efficiency and productivity of the swine industry. Hence, screening for gilts with increased reproductive longevity potential in early life is of great research interest.

The selection for the breeding of modern swine involves a comprehensive approach that combines various techniques [8], such as phenotypic selection [9], crossbreeding [10], genomic selection [11], and artificial insemination [12]. It has been widely reported that the microbiota is associated with host physiological function [13–15], pregnancy [16, 17], and reproductive performance [18]. Interestingly, recent research has indicated that the gut and vaginal microbiota could affect estrus return in post-weaning sows through its effect on sex hormones [19, 20]. These studies suggest that the microbiota serve as a potential method for screening for sows with increased reproductive efficiency traits. Importantly, microbes dynamically change within different growth or physiological periods. Thus, understanding how the microbiota varies within specific stages throughout the swine life cycle helps develop targeted strategies to improve the health and productivity of pigs. It has been illustrated that the composition of the swine fecal microbiome is altered between the pregnancy and lactation stages [17]. Additionally, our previous study revealed the longitudinal alternation of the swine gut microbiome from birth to market [21]. The temporal dynamic analysis of the female pig’s fecal microbiome from early life to the first parity has also been elucidated [22]. Nevertheless, how the vaginal and rectal microbiota change over successive parities is unclear.

Therefore, in the present study, we screened sows with reproductive longevity that could complete multiple parities from a total of 48 female pigs. To characterize sows with high reproductive efficiency in early life, we compared and identified their microbial characteristics with those of culled sows experiencing reproductive failures. Further, we profiled the vaginal and rectal microbiome of sows with longevity potential from the breeding stage to lactation throughout four successive parities. The relationships between different parities, microbes, and reproductive performance were investigated in this study. Our analyses broaden the understanding of the microbiome dynamics throughout four parities and provide a basis for future studies exploring microbe-targeted strategies to promote pig reproductivity.

## Methods

### Animal husbandry

Gilts/sows were cared for and managed according to the protocol guideline, which was approved by the University of Arkansas Institutional Animal Care and Use Committee #21044.

#### Gilt development

Three groups of 16 gilts (PC1050) were selected at 9 weeks of age and transferred from multipliers farm to the University of Arkansas Wean to the Finisher Research unit. Upon arrival, individual gilt body weight was recorded, and rectal and vaginal swabs were collected. Gilts were housed in a total slot floor pen, and each pen (1.5 m × 3.0 m) was equipped with a single-hole feeder and a cup waterer. Gilts had ad libitum access to feed and water during development periods. The diet changed according to different phases (Table S1). They were placed on a three-stage feeding program as follows: phase 1 (22.7 to 56.8 kg), phase 2 (56.8 to 90.8 kg), and phase 3 (90.8 to 131.7 kg). Gilts were fully fed until they were moved into the breeding unit (stall) at 26 weeks of age. Boar stimulation was initiated starting at week 23 and continuing through week 25 by driving gilt to an empty pen between two pens of 2 boars/pen in another room. Starting with estrus detection from week 23 of age through d 182 of week 26, all gilts remained in pens with full feed.

#### Gilt breeding

At the initiation of week 26 (d 182), all gilts were transferred to the breeding facility and were placed in individual gestation stalls (2.2 m × 56 cm). At the same time, boar exposure (fence) was continued until week 27 (d 189) when altrenogest (MATRIX^®^, MERCK Animal Health USA, Rahway) was initiated. Once gilts were moved to the breeding facility at week 26, all gilts were fed 2.7 kg/d for one week to allow gilts to adjust to the stalls. That was followed by simulated full feeding until breeding to ensure maximum ovulation. During breeding on week 30, their intake level was reduced to 1.8 kg/d. Note that all gilts were placed on MATRIX^®^ starting on d 189 of week 27. On d 203, MATRIX^®^ was removed so gilts would cycle for breeding during week 30. The intent was to breed on their second heat cycle.

#### Sow breeding

After lactation, sows were transferred back to the breeding facility to allow rebreeding for subsequent cycles, and 1.8 kg/d of gestation diets were offered during breeding.

#### Gestation

Gilts and sows were bred during week 30, and feed was again reduced to 1.8 kg/d for the breeding period. Soon after breeding, their body condition was evaluated using a caliper (Pipestone Veterinary Services, Pipestone, MN, USA), and the feed level was adjusted accordingly. From 35 to 42 d after breeding until they were moved to lactation on d 110 of gestation, gilts were placed in a pen system with an automated Gestal (JYGA technologies, QC, Canada) feeding system to program feed intake based on their body condition score measured before pen system placement.

#### Lactation

When they moved to the farrowing facility, gilts and sows were fed lactation diets. The amount of feed remained at the levels they were fed during gestation, approximately 1.8 kg, with twice-daily feeding until parturition. Within 24 h of parturition, gilts and sows were fed at least two times daily and fed to appetite. Sows were allowed to nurse their progeny for 21 d, and they were transferred to gestation stalls to allow rebreeding for subsequent cycles.

#### Data collection

The weight of individual piglets at birth and weaning was measured, and the number of born alive and weaned piglets per litter was recorded.

### Sample collection, DNA extraction, and sequencing

Rectal and vaginal swabs (Puritan^®^Opti-Swab^®^, Guilford, Maine, USA) were collected from 48 sows repeatedly starting at 9 weeks of age, breeding (d 0), and d 110 of gestation of parity 1, and again on d 0 and d 110 of gestation for following parties up to four parities. To avoid cross-contamination, the exterior area of the vulva was cleaned before inserting the swabs. The collection was operated by two people, with one person opening the vulva and a second person performing the collection. Vaginal swabs were collected before rectal swabs. These swabs were stored at −80 °C ultra-low freezer for further analysis. According to the aim of this study, a total of 186 swabs, including 93 rectal and 93 vaginal swabs, were selected for DNA extraction.

Bacterial DNA was extracted using the PowerLyzer PowerSoil Kit (Qiagen, Hilden, Germany) according to the manufacturer’s instructions. The NanoDrop (Thermo Fisher Scientific, Wilmington, DE, USA) was used to detect DNA concentrations. All the DNA was diluted to 10 ng/µL with nuclease-free water. The DNA library was constructed according to the previous protocol [23]. The V4 region of bacterial 16S rRNA gene was amplified using universal primers (Forward: 5′-GTGCCAGCMGCCGCGGTAA-3′, and Reverse: 5′-GGACTACHVGGGTWTCTAAT-3′) with attached barcoded index and Illumina adapters. To verify and normalize the accurate size of the PCR product, agarose gel electrophoresis was performed first, and then the SequalPrep Normalization Plate Kit (Invitrogen, Carlsbad, CA, USA) was used. The normalized amplicon concentrations were detected using a Qubit fluorometer (Thermo Fisher Scientific, Waltham, MA, USA). All the sample DNA were pooled together in equal volume. To verify the quality and quantity of the pooled amplicons, the DNA was measured with an Agilent Bioanalyzer 2100 (Agilent, Santa Clara, CA, USA) and quantitative RT-PCR (Eppendorf, Westbury, NY, USA) assay through a KAPA Library Quantification Kit (Kapa Biosystems, Woburn, MA, USA).

Illumina MiSeq 2 × 250 bp paired-end sequencing (MiSeq Reagent Kit v2, 500 cycles, 20% PhiX) was used to sequence pooled amplicons. Negative controls were set for the processing of DNA extraction and PCR amplification. A mock community (ZymoBIOMICS Microbial Community Standard; Zymo, Irvine, CA, USA), including a defined mixture of microbial genomic DNAs with known proportions, was used as a positive control in each MiSeq run.

### Microbiome data analysis

The Illumina MiSeq fastq reads downloaded from the Illumina BaseSpace^®^ website and were analyzed in the QIIME2 platform [24]. The Deblur program [25] was used to process the raw data as described below. First, raw forward and reverse reads were merged by “qiime vsearch merge-pairs”. Second, the merged reads were filtered by “qiime quality-filter q-score” (--p-min-quality 30 --p-quality-window 5). Third, the filtered reads were trimmed by “qiime deblur denoise-16S” (--p-left-trim-len 3 --p-trim-length 250). Deblur-denoised sequences were assigned to amplicon sequence variants (ASVs) at single-nucleotide resolution in this study. To minimize the effects of sequencing depth on subsequent analysis, all samples were rarefied to the lowest reads by “qiime diversity core-metrics-phylogenetic”. The classification of the ASVs was based on the training database by “qiime feature-classifier classify-sklearn”. The training database was downloaded from the silva (https://www.arb-silva.de/no_cache/download/archive/qiime) and converted from 16S to V4 region. The positive control in this study yielded high-quality reads and showed consistent relative abundances of each bacterial ASV, as suggested by mock community analysis. Alpha and beta diversity analysis and analysis of similarity (ANOSIM) were also conducted in the QIIME2 platform by “qiime diversity alpha” and “qiime diversity beta”. Beta diversity was evaluated using Bray-Curtis distances to demonstrate the dissimilarity between groups. The PICRUSt2 plugin in the QIIME2 platform was used to predict the bacterial function. The generated KEGG orthologous abundance sheet was used for further analysis.

### Statistical analysis

All data were analyzed and visualized in R (version 4.3.1). For microbiome data, we used non-parametric tests. The Mann-Whitney U test was used to detect significant differences between 2 groups, while the Kruskal-Wallis H was used to detect significant differences for multiple groups. The Benjamini–Hochberg false discovery rate (FDR) analysis was used for multiple comparison correction. For reproductive performance data, we used parametric tests. The *t*-test was used to calculate the difference between two groups while the one-way ANOVA was used to detect significant differences for multiple comparisons with Fisher’s LSD test. The *P* value < 0.05 was considered statistically significant.

RandomForest in R was used to predict the ASVs associated with longevity and pregnancy. The relative abundance of the top 1000 ASVs was input as the training database for the random forest model. The optimal markers between the U4P and non-U4P groups based on repeated cross-validation of the random forest model were selected according to ranked Mean Decrease Accuracy values using the AUCRF and randomForest package in R. The performance of optimal markers was evaluated by AUC value using the pROC package in R. Also, random forest was used to identify the bacterial ASVs correlated with reproductive performance according to %IncMSE in the default settings.

The clusterProfiler package in R was used to enrich the KEGG pathways according to the selected significantly different genes. FDR analysis was used to correct the *P* value.

The distinguished bacterial ASVs in each parity and continuously different bacterial ASVs between d 0 and d 110 were identified by linear discriminant analysis effect size (LEfSe) with LDA value > 3.5 and *P* value < 0.05 using the OmicStudio tools (https://www.omicstudio.cn/tool).

The bacterial correlation network was calculated by using the Spearman method and the igraph package in R first. The code and edge information were exported and visualized in Gephi (version 0.10.1).

The Mantel test was used to observe the matrix-based correlation between microbiome and reproductive performance using the linkET package in R.

## Results

### Pregnancy description of sows in four parities

**Fig. 1:**
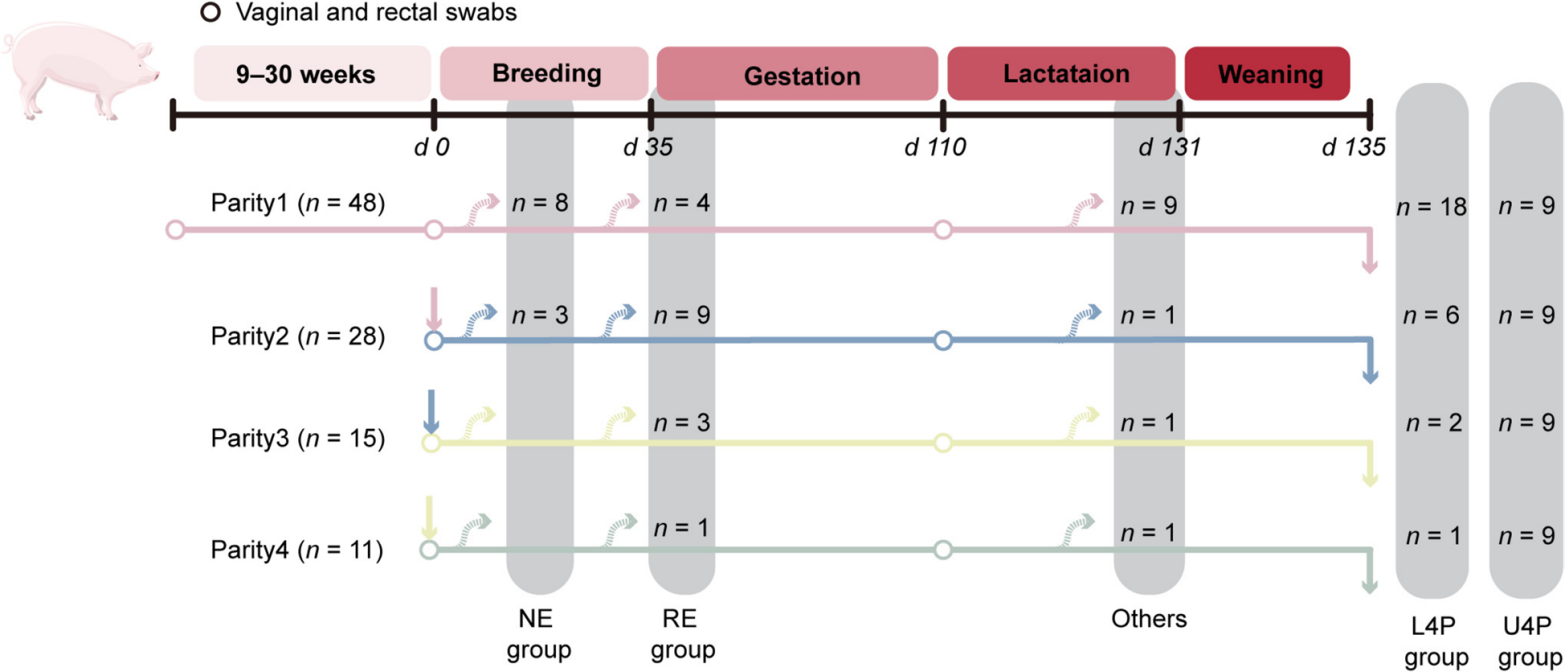
Experimental design

In this study, 48 sows were screened for reproductive longevity according to whether they successfully completed four farrowing cycles (Fig. 1). Only nine out of the 48 sows completed all four parities (U4P group). In each parity, sows that were pregnant in the given parity but were culled in the following parity were classified into the L4P group. In the breeding stage, eight and three sows were culled due to no estrus (NE group) in parity 1 and parity 2, respectively. There were four, nine, three, and one sows that displayed the estrus cycle but failed to conceive (RE group) in parity 1, 2, 3, and 4, respectively. In addition, a total of nine, one, one, and one sows in parity 1, 2, 3, and 4, respectively, were removed due to abortion and other reasons in this study.

### Bacterial signatures differentiate sows with longevity potential in the early breeding stage of the primiparous cycle

**Fig. 2:**
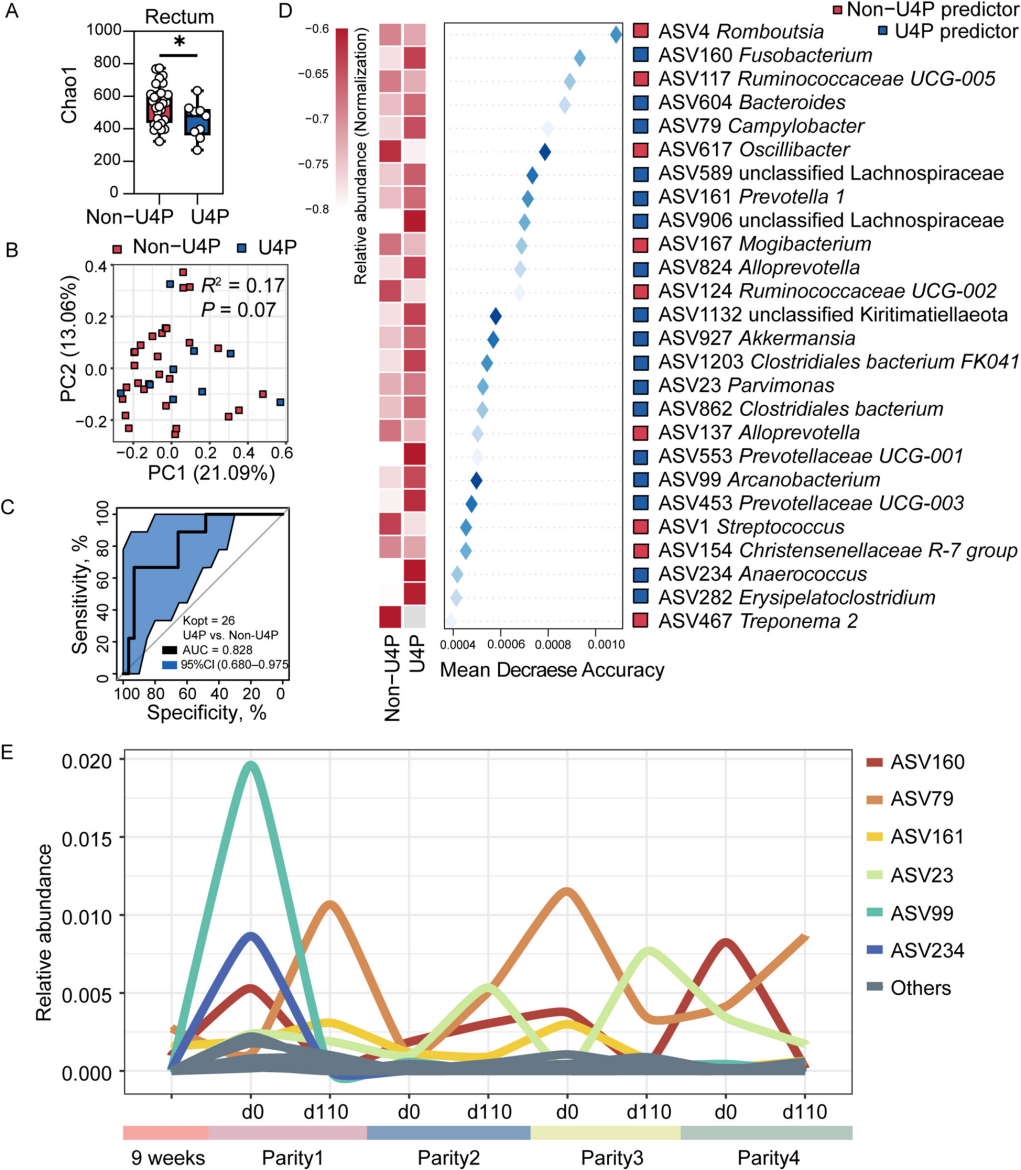
The rectal distinguished bacteria of U4P group in the early breeding stage of first parity. **A** Boxplot of the Chao1 index between U4P and non-U4P group in the rectum. **B** PCoA plots based on the Bray-Curtis distances in the rectum. **C** The ROC curve of the random forest model based on optimal 26 ASVs in the rectum. **D** The optimal 26 predictors between the U4P and non-U4P groups in the rectum. Left panel: The relative abundance of bacterial ASVs. Right panel: The repeated cross-validation of the random forest model according to Mean Decrease Accuracy values. **E** The curve chart of distinguished ASVs of U4P in a longitudinal period in the rectum. All values in boxplot are expressed as mean ± SEM. The asterisks mean statistically significant difference: ^*^P value < 0.05

**Fig. 3:**
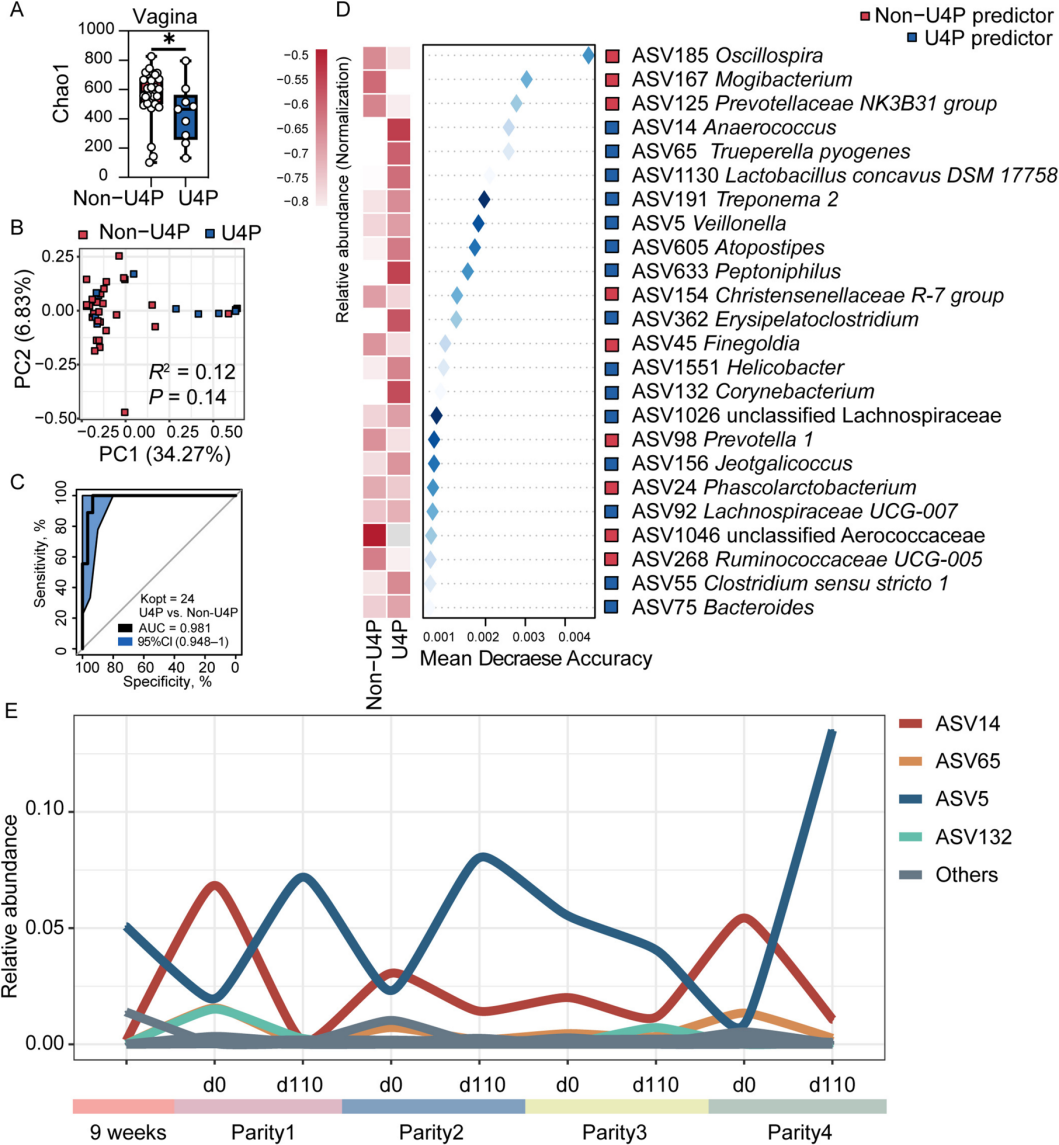
The vaginal distinguished bacteria of U4P group in the early breeding stage of first parity. **A** Boxplot of the Chao1 index between U4P and non-U4P group in the vagina. **B** PCoA plots based on the Bray-Curtis distances in the vagina. **C** The ROC curve of the random forest model based on optimal 24 ASVs in the vagina. **D** The optimal 24 predictors between the U4P and non-U4P groups in the vagina. Left panel: The relative abundance of bacterial ASVs. Right panel: The repeated cross-validation of the random forest model according to Mean Decrease Accuracy values. **E** The curve chart of distinguished ASVs of U4P in a longitudinal period in the vagina. All values in boxplot are expressed as mean ± SEM. The asterisks mean statistically significant difference: ^*^P value < 0.05

First, we observed different bacterial diversity (Fig. S1) and composition (Fig. S2) in sows with different breeding outcomes. To compare and differentiate the microbiome of sows with reproductive longevity (U4P) to those non-U4P group counterparts, including the L4P, NE, and RE, the breeding (d 0) swabs samples of the first parity were analyzed. As shown by the Chao1 index (Figs. 2A and 3A), the U4P group exhibited decreased richness compared to the non-U4P group in both the rectum and vagina. Additionally, there was a tendency to have a different community structure (*P* = 0.07) between U4P and non-U4P by analyzing the β-diversity based on Bray-Curtis distances in the rectum (Fig. 2B). However, the difference was limited in the vagina between groups (Fig. 3B).

Next, a RandomForest classifier was built and tested to identify distinguished bacterial ASVs in the early breeding stage of the first parity between U4P and non-U4P. Here, we identified 26 ASVs differentially represented between the two groups in the rectum. Additionally, these 26 ASVs predicted sow reproductive longevity with an AUC value of 0.828 (Fig. 2C). Among them, 17 ASVs (e.g., ASV604_*Bacteroides*, ASV161_*Prevotella 1*, ASV927_*Akkermansia*) were enriched in the U4P group, whereas the other 9 bacteria (e.g., ASV4_*Romboutsia*, ASV117_*Ruminococcaceae UCG-005*) were in the non-U4P group (Fig. 2D). In the vagina, 24 ASVs were differentiated between the two groups and predicted sow longevity with an AUC value of 0.981 (Fig. 3C). Among them, 15 ASVs (e.g., ASV14_*Anaerococcus*, ASV1130_*Lactobacillus concavus*, ASV5_*Veillonella*) were more abundant in U4P while the remaining 9 ASVs (e.g., ASV185_*Oscillospira*, ASV167_*Mogibacterium*) were greater in the non-U4P (Fig. 3D). Additionally, we found that the bacterial predictors in the vagina represented a more complex inter- and intra-group interaction between U4P and non-U4P groups compared to in the rectum (Fig. S3).

Of note, we tracked the relative abundance of these bacterial predictors in the U4P group throughout the four parities. Interestingly, in the rectum, ASV160_*Fusobacterium*, ASV79_*Campylobacter*, ASV161_*Prevotella 1*, ASV23_*Parvimonas* seemed to persist with higher relative abundances throughout the experiment, while ASV99_*Arcanobacterium* and ASV234_*Anaerococcus* appeared only showed a high relative abundance on d 0 of the first parity (Fig. 2E). In the vagina, ASV14_*Anaerococcus* and ASV5_*Veillonella* persisted throughout all 4 parities (Fig. 3E).

### Sows with longevity potential exhibited different bacterial functions

**Fig. 4:**
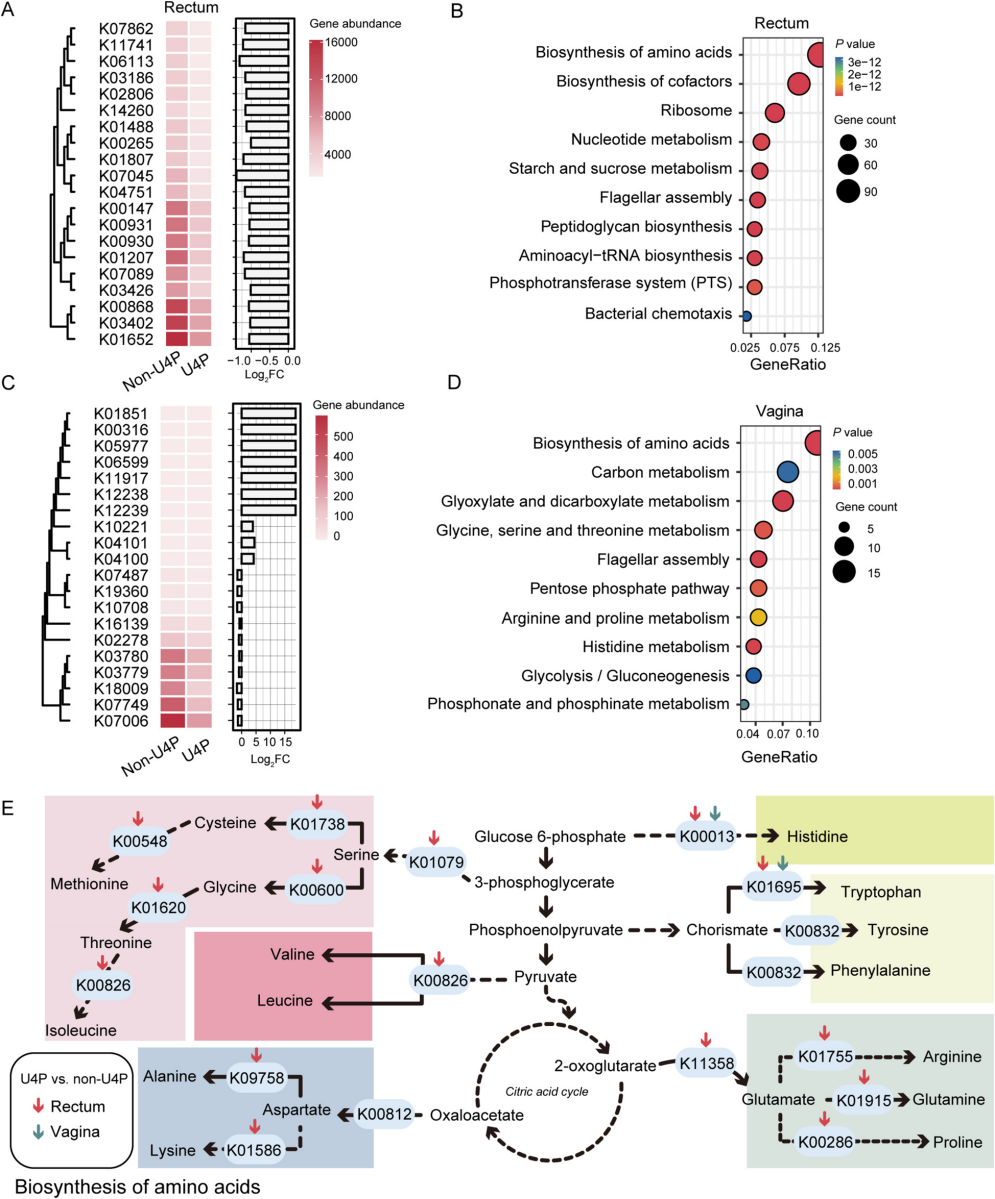
Predicted bacterial function differences between the U4P and non-U4P groups. **A **and **C** The top 20 significantly different KEGG orthologous genes between U4P and non-U4P groups in the rectum (**A**) and vagina (**C**). Left panel: The heatmap depicting the gene abundance. Right panel: The bar plot illustrating the Log_2_FC (U4P/non-U4P). **B** and **D** The KEGG enriched pathway of discriminant genes (*P* value < 0.05) in the rectum (**B**) and vagina (**D**). **E** The biosynthesis of amino acids pathway. The dotted line indicates omitted steps. The red/blue downward arrow indicates that the gene has a significant decrease in U4P compared to non-U4P in the rectum/vagina

To further explore how bacteria affect sow longevity, we utilized PICRUSt2 to predict bacterial function. First, we have depicted the top 20 significantly different KEGG orthologous (KO) genes in the rectum (Fig. 4A) and vagina (Fig. 4C). In the rectum, the U4P group had decreased abundance of genes such as acetolactate synthase (K01652) and transcriptional regulator of arginine metabolism (K03402). In the vagina, U4P had an increased abundance of genes, such as salicylate biosynthesis isochorismate synthase (K01851), and decreased abundance of genes, such as formyl-CoA transferase (K07749). Then, the enrichment pathway of these discriminant KO genes illustrated the top 10 pathways respectively in the rectum (Fig. 4B) and vagina (Fig. 4D). Interestingly, the biosynthesis of amino acids pathway (ko01230) was altered between groups in both niches. Correspondingly, we further mapped the genes responsible for the synthesis of amino acids in the pathway (Fig. 4E). The U4P exhibited a lower abundance of tryptophan synthase alpha chain (K01695) and histidinol dehydrogenase (K00013), responsible for tryptophan and histidine synthesis, in both the rectum and vagina.

### Longitudinal investigation of bacterial communities for breeding sows throughout four parities

Another goal of this study was to understand how the microbiota of the sows with reproductive longevity changes across the four parities. To do so, we investigated the microbiome from the nine sows in the U4P group throughout the four parities. Firstly, we examined the relative abundance of the top 15 genera at nine-time points (9 weeks old gilts, d 0 and d 110 of gestation for each parity) throughout the four parities in the rectum (Fig. S4A) and vagina (Fig. S4B), among which seven genera, including *Clostridium sensu stricto 1*, *Turicibacter*, *Streptococcus*, *Anaerococcus*, *Terrisporobacter*, *Porphyromonas*, and *Romboutsia* were shared. In more detail, the top 20 ASVs are also illustrated. ASV2_*Turicibacter*, ASV1_*Streptococcus*, and ASV3_*Lactobacillus* were the top 3 ASVs in the rectum (Fig. S4C), while ASV5_*Veillonella*, ASV7_unclassified Pasteurellaceae and ASV13_ *Actinobacillus rossii* were the top 3 ASVs in the vagina (Fig. S4D).

**Fig. 5:**
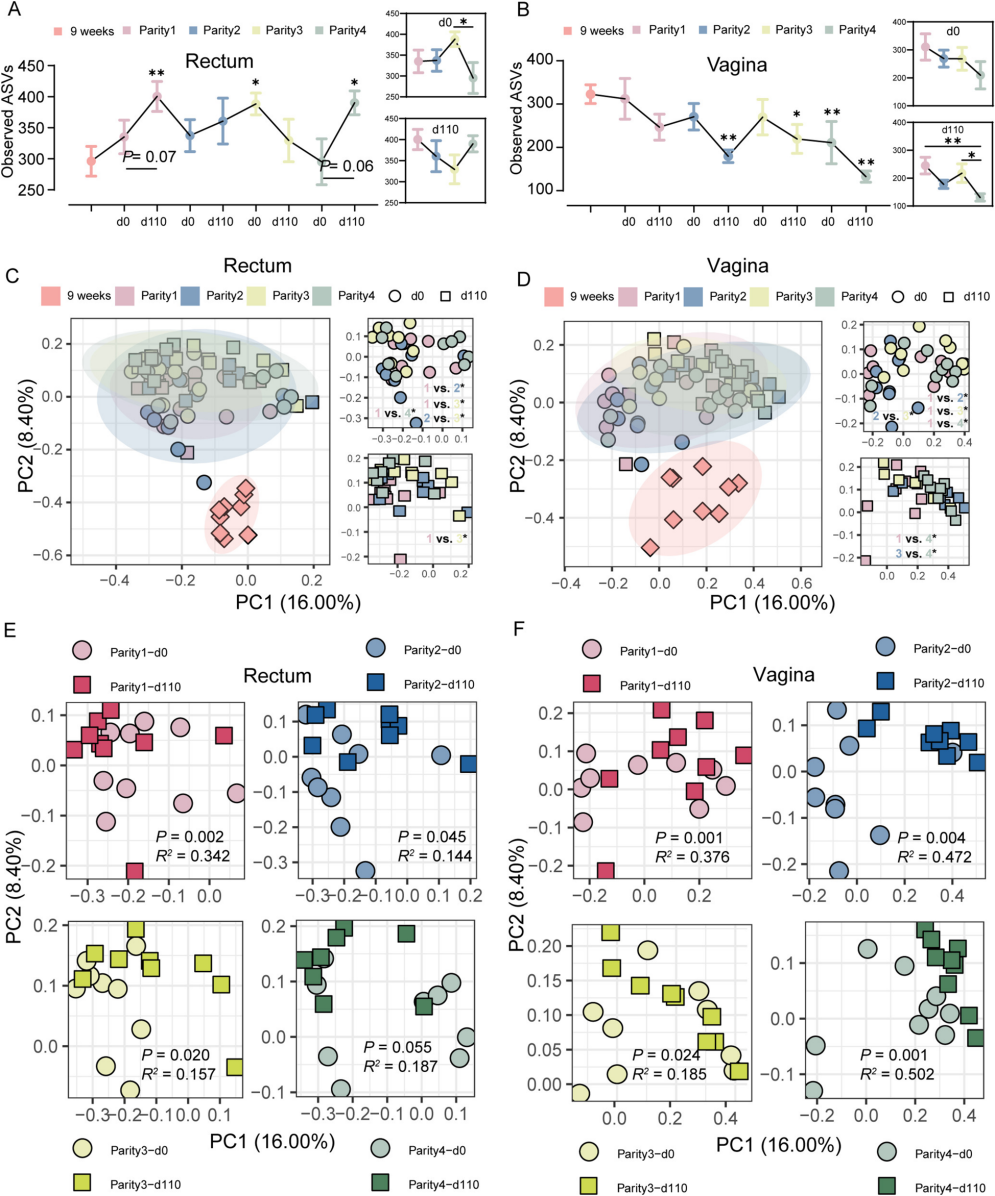
Dynamic changes of bacterial diversity in the U4P group through four parities. Boxplot illustrating observed ASVs in the d 0 and d 110 through 4 parities in the rectum (**A**) and vagina (**B**). PCoA plots illustrating the bacterial composition between 4 parities based on the Bray-Curtis distances in the rectum (**C**) and vagina (**D**). PCoA plots illustrating the bacterial composition between d 0 and d 110 in each parity based on the Bray-Curtis distances in rectum (**E**) and vagina (**F**). Asterisks mean statistically significant difference: ^*^P value < 0.05; ^**^P value < 0.01

Next, we characterized the bacterial diversity from estrus (d 0) to d 110 of gestation in all four parities. Interestingly, the observed ASVs on d 110 were increased compared to d 0 of gestation in each parity except parity 3 in the rectum (Fig. 5A). In contrast, a controversial response pattern was observed in the vagina. Moreover, vaginal observed ASVs showed a continued downward trend throughout parity in this study (Fig. 5B). It was worth noting that the observed ASVs of each parity decreased from d 0 to d 110 of gestation. Still, when entering d 0 of the next parity, the diversity increased slightly. This may be related to changes in the reproductive tract of sows before and after parturition. Based on the principal coordinate analysis (PCoA) plots with Bray-Curtis distances, the bacterial membership and structure in gilts at nine weeks of age showed significant differences compared with the subsequent eight-time points in both the rectum and vagina (Fig. 5C and D). When comparing the differences of parity on diversity on d 0 and d 110 of gestation, we found that there were significant differences in d 0 of gestation between each parity, but less so on d 110 in both the rectum and vagina (Fig. 5C and D). To compare the difference more clearly between d 0 and d 110 for each parity, we performed a more detailed analysis. Significant shifts were observed between d 0 and d 110 for each parity in both the rectum and vagina (Fig. 5E and F).

### Different bacteria between four parities

**Fig. 6:**
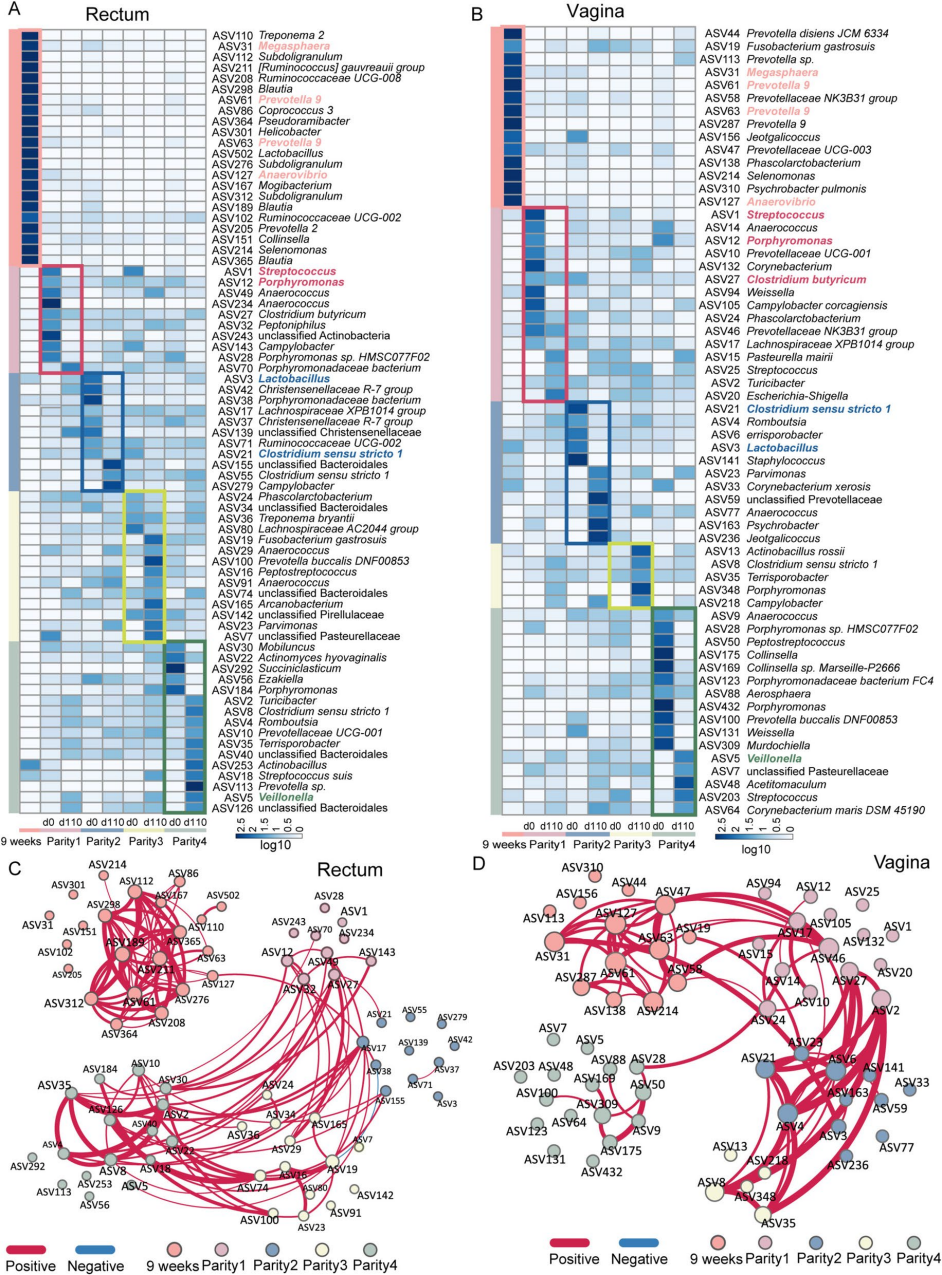
The distinguished bacterial ASVs in each parity and their correlation. Heatmap showing parity-associated bacterial ASV identified by LEfSe (LDA > 3.5 and *P* value < 0.05) analysis in the rectum (**A**) and vagina (**B**). The average relative abundance is normalized in a log scale. The network of distinguished ASVs correlation between each parity in the rectum (**C**) and vagina (**D**). The correlations between ASV were shown based on the Spearman rho over 0.4 or less than −0.4 and adjusted *P* value < 0.05

 To identify bacterial ASVs enriched between different parities, LEfSe analysis was used, and the relative abundance of these ASVs was visualized using a heat map (Fig. 6A and B). Interestingly, we observed certain bacterial ASVs represented each stage in both the rectum and vagina. For example, ASV31_*Megasphaera*, ASV61_*Prevotella 9*, and ASV127_ *Anaerovibrio* were all enriched in the rectum and vagina of U4P group sows at nine weeks of age. ASV1_*Streptococcus*, ASV12_*Porphyromonas*, and ASV27_*Clostridium butyricum* were enriched in parity 1. ASV3_*Lactobacillus* and ASV21_ *Clostridium sensu stricto 1* were enriched in parity 2. ASV5_*Veillonella* was enriched in parity 4. Network analysis also illustrated the interaction between parity-associated bacterial ASVs. Among them, the nine-week-enriched ASVs are less connected with the ASVs of the other three parties in the rectum (Fig. 6C). However, in the vagina, enriched ASVs in parity 4 are less connected with the ASVs enriched in other parities (Fig. 6D). Together, these data illustrate ASVs shared between the rectum and vagina at different stages and indicate that sows have unique bacterial interactions at different stages.

### Different bacteria between breeding and parturition stages

**Fig. 7:**
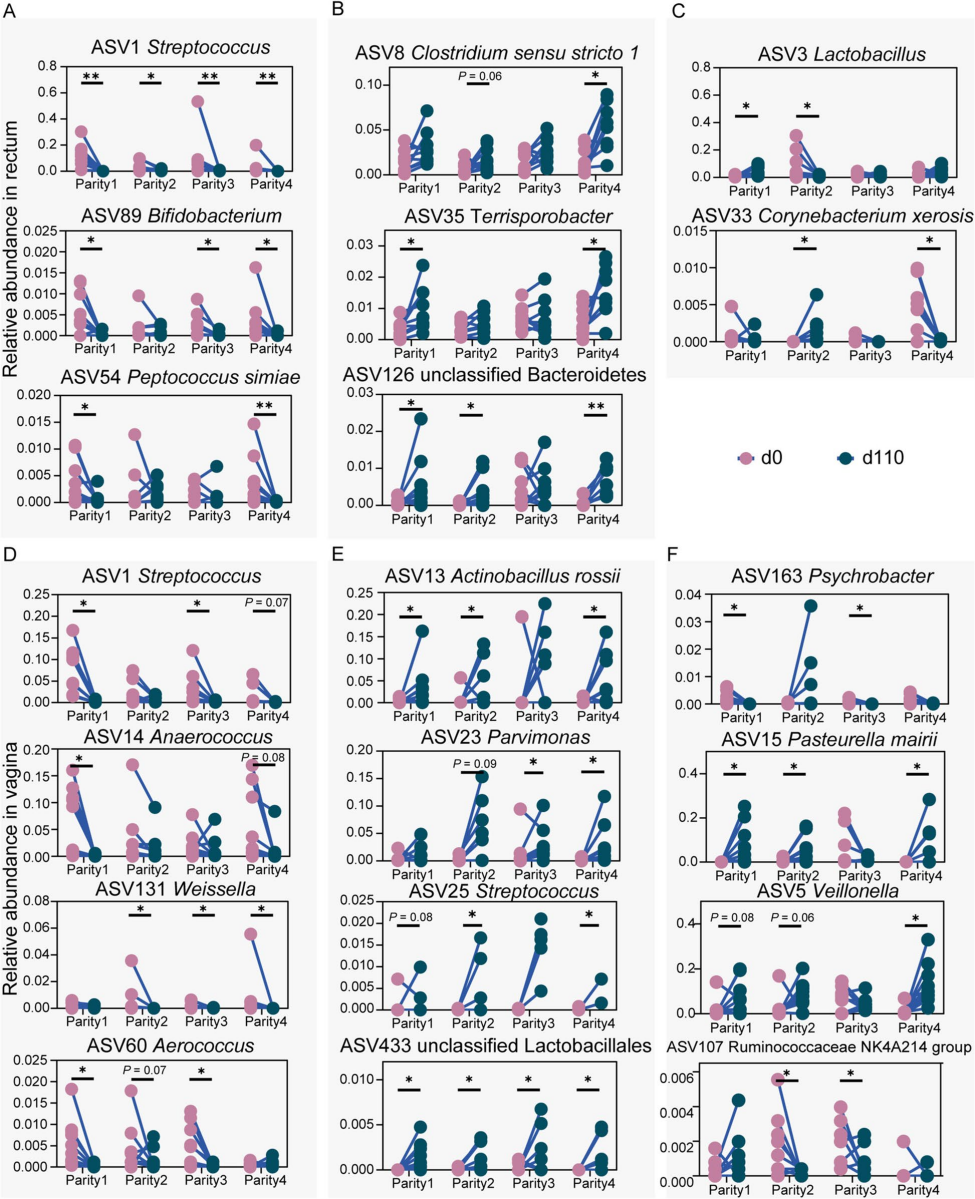
The continuously different bacterial ASVs between d 0 and d 110 through four parities. Line chart illustrating the relative abundance of continuously decreased ASVs (**A**), increased ASVs (**B**), and mixed ASVs (**C**) from d 0 to d 110 in the four parities in the rectum. Line chart illustrating the relative abundance of continuously decreased ASVs (**D**), increased ASVs (**E**), and mixed ASVs (**F**) from d 0 to d 110 in the four parities in the vagina. The asterisks mean statistically significant difference: ^*^P value < 0.05; ^**^P value < 0.01

 To compare bacterial patterns between the breeding (d 0) and parturition (d 110) stages within each parity, LEfSe analysis was used to identify bacteria that significantly changed between d 0 and d 110 in two or more parities. Next, we classified those ASVs into three groups: declined (bacteria decreased significantly from d 0 and d 110 in two or more parities), elevated (bacteria increased significantly from d 0 to d 110 in two or more parities), and mixed (bacteria decreased or increased significantly from d 0 to d 110 in two or more parities). ASV1_*Streptococcus*, ASV89_*Bifidobacterium*, and ASV54_*Peptococcus simiae* in the rectum (Fig. 7A), while ASV1_*Streptococcus*, ASV14_*Anaerococcus*, ASV131_*Weissella* and ASV60_*Aerococcus* in the vagina (Fig. 7D) declined from d 0 to d 110 of gestation. Meanwhile, ASV8_*Clostridium sensu stricto 1*, ASV35_*Terrisporobacter* and ASV126_ unclassified Bacteroidetes in the rectum (Fig. 7B), and ASV13_*Actinobacillus rossii*, ASV23_*Parvimonas*, ASV25_*Streptococcus* and ASV433_unclassified Lactobacillales (Fig. 7E) were elevated from d 0 to d 110 of gestation in the vagina. ASV3_*Lactobacillus* and ASV33_*Corynebacterium xerosis* from the rectum (Fig. 7C), and ASV163_*Psychrobacter*, ASV15_*Pasteurella mairii*, ASV5_*Veillonella* and ASV107_*Ruminococcaceae NK4A214 group* from vagina had inconsistent response patterns and were classified as mixed (Fig. 7F). Taken together, these results suggest that sows have different bacterial dynamics in the breeding and parturition stages.

### Sows with longevity potential demonstrate better reproductive performance

**Fig. 8:**
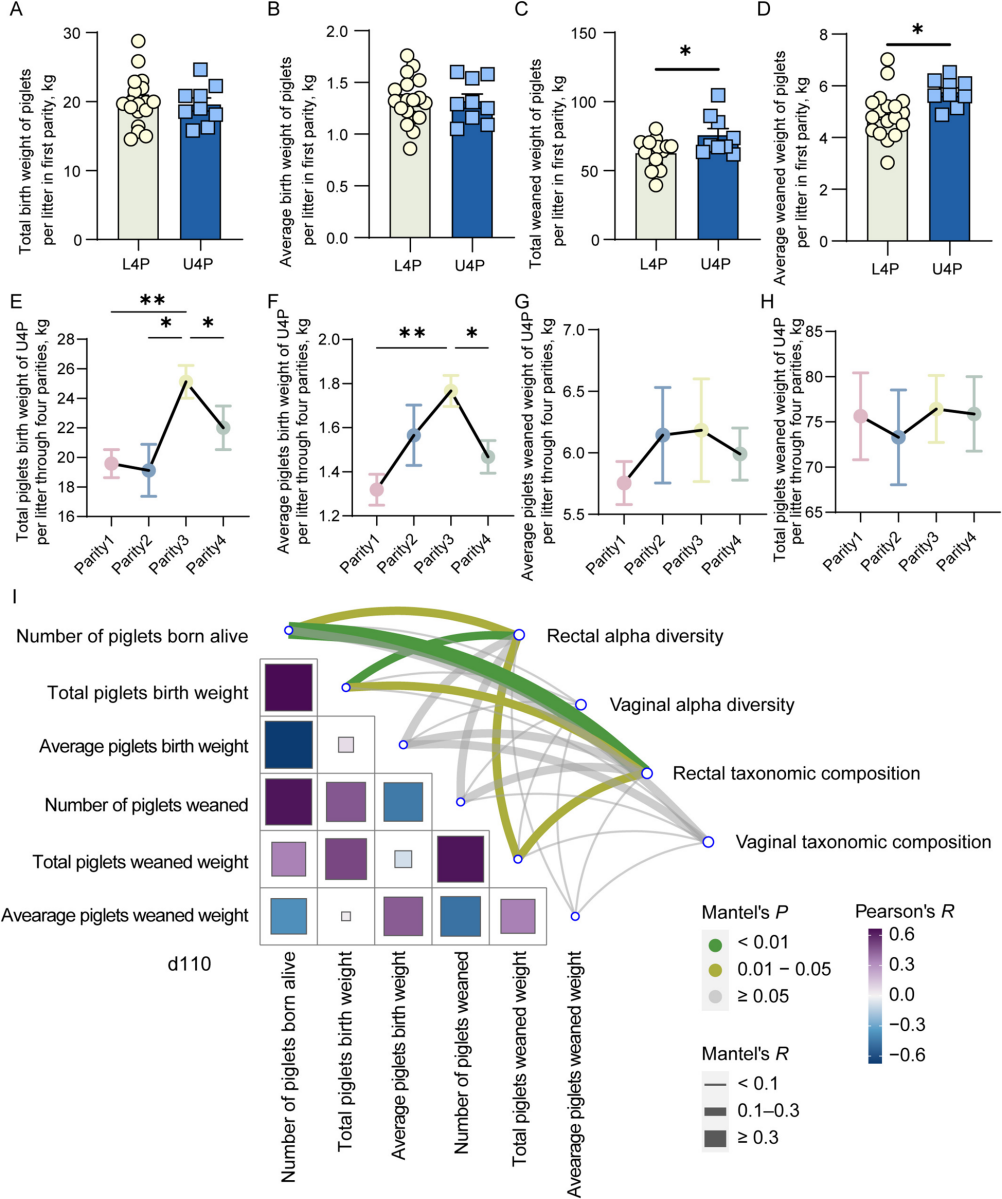
The better reproductive performance of U4P group. Bar chart illustrating the total birth weight of piglets (**A**), average birth weight of piglets (**B**), total weaned weight of piglets (**C**), average weaned weight of piglets (**D**) per litter between L4P and U4P in the first parity. Line chart illustrating the total birth weight of piglets (**E**), average birth weight of piglets (**F**), total weaned weight of piglets (**G**), average weaned weight of piglets (**H**), per litter of U4P groups through four parities. The asterisks mean statistically significant difference: ^*^P value < 0.05; ^**^P  value < 0.01. The Mantel *t*-test showing the correlation between reproductive performance and bacterial diversity and composition d 110 (**I**). The line between the two points represents the relationship between reproductive performance and bacterial characteristics, the thickness represents the size of Mantel’s R, and the color represents the range of Mantel’s *P* values

 We compared the reproductive performance, including six indicators (number of born alive piglets, total birth weight of piglets, average birth weight of piglets, number of weaned piglets, total weaned weight of piglets, and average weaned weight of piglets) between the L4P and U4P groups in the first parity. No significant difference existed in the number of born alive and weaned piglets (Fig. S5A and S5B). We did not observe significant differences in the total and average birth weight of piglets (Fig. 8A and B). Interestingly, the total and average weaned weight of piglets per litter in the U4P was significantly higher than in L4P (Fig. 8C and D). Following the change in reproductive performance of the U4P group throughout the four parities, the results showed that the total and average birth weight of piglets (Fig. 8E and F) both increased continuously from parity 1 to parity 3, but decreased from parity 3 to parity 4. Furthermore, no difference was observed for the total and average weaned weight of piglets between the four parities (Fig. 8G and H). Also, there was no significant difference in the number of born alive and weaned piglets between the four parities (Fig. S5C and S5D). These results revealed that the U4P was much more capable of nursing their progeny when compared to L4P in the first parity.

### Bacteria associate with reproductive performance

The Mantel *t*-test examined the correlation between reproductive performance and bacteria. On d 0, we did not observe a significant correlation between bacteria and reproductive performance (Fig. S5E). On d 110, rectal alpha diversity and taxonomic composition correlated significantly with the number of piglets born alive, total birth weight of piglets, and total weaned weight of piglets (Fig. 8I).

Next, this study explored whether bacteria associated with pregnancy and longevity correlated with sows’ reproductive performance in the U4P group. In the rectum (Fig. S6A), ASV160_*Fusobacterium* correlated negatively with the average birth weight of piglets., and the number of weaned piglets showed a negative correlation with ASV906_unclassified Lachnospiraceae and a positive correlation with ASV99_*Arcanobacterium*. In the vagina, ASV362_*Erysipelatoclostridium* correlated positively with the average weaned weight of piglets, and ASV1551_*Helicobacter* correlated negatively with the total birth weight of piglets (Fig. S6B).

**Fig. 9:**
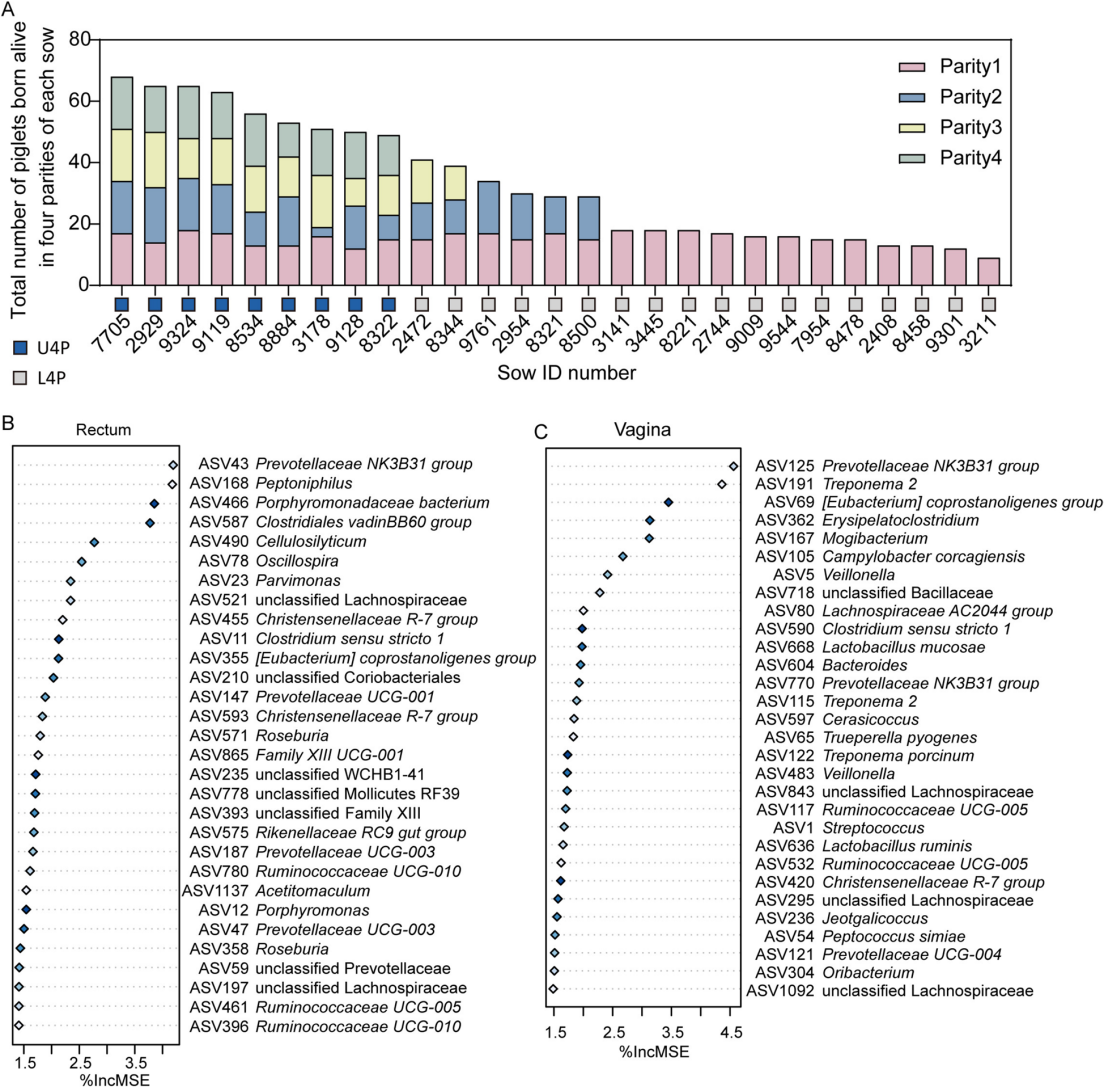
The prediction of the reproductive performance-associated bacterial ASVs in the first early breeding stage. The stacked column chart (**A**) exhibiting the total number of piglets born alive in the four parities of each sow. Total number of piglets-related top 30 ASVs on d 0 of first parity using the regression-based random forest in the rectum (**B**) and vagina (**C**)

The total number of born-alive piglets is considered an important indicator of reproductive performance in sow production. To compare and summarize the reproductive potential of each individual sow, we calculated the total number of piglets in four parities for each sow. The results showed that the sow tagged 7705 in the U4P group had the highest total number of born alive piglets (Fig. 9A). RandomForest was used to predict the bacteria associated with the total number of piglets in four parities. Here, we listed the top 30 bacterial ASVs in the rectum and vagina (Fig. 9B and C). Interestingly, we found that *Prevotellaceae NK3B31 group* was the top predictor in both the vagina and rectum.

## Discussion

### Longevity-associated bacteria in reproduction

The sow is the most important commodity in the swine production sector. Reproductive failure can reduce sow productivity and decrease the profitability of production [26]. To be more specific, non-estrus in breeding sows may lead to culling and removal, affecting the productivity of the pig industry [27]. Previous studies have shown that both the gut and vaginal microbiota played important roles in estrus return of sows [19, 20]. Bacterial species *L. reuteri* and *Prevotella* spp. in fecal samples were identified to be associated with estrus return and regulating estrogen biosynthesis. However, sows in that study were distributed from parity 4 to 7, which was unsuitable for explaining the interrelationship of longevity and microbiota. Current production practice is to retire the sow after four parity cycles as sows in parity 5 or higher have decreased ovulation and fertilization rates and increased embryonic mortality and pregnancy loss [26]. In order to understand the correlation between longevity and microbiota, we followed gilts throughout four parities and observed specific bacteria in the breeding stage of the first parity between gilts that could or could not finish four parities. We then identified these bacterial predictors for the sows with high reproductive efficiency by examining the microbiome over eight-time points through four parities. Like the previous study, sows that successfully showed estrus had a lower alpha diversity compared to the others facing reproductive failures in our analysis. *Akkermansia* and *Lactobacillus*, which have been widely recognized to exhibit probiotic properties, were identified in the present study. Interestingly, we identified some pathogenic bacteria with low abundance at the breeding stage of the primiparous cycle, such as *Trueperella pyogenes* [28] and *Campylobacter* [29], in the vagina and rectum. We speculate that the reddening and swelling of the vulva and mucus secretion in estrus may lead to the growth of some pathogenic bacteria [30, 31]. However, the correlation between pathogens with low relative abundance and longevity remains unclear and investigated. Furthermore, some bacteria, including *Veillonella* and *Anaerococcus*, whose functions in reproductive health have rarely been reported in pigs, were predictive of reproductive longevity. Further validation is needed to determine whether these bacteria could be used to predict sows with longevity traits in reproduction. Moreover, the prediction of bacterial function revealed for the first time that sows with longevity potential in the early breeding stage exhibited lower gene abundance for the synthesis of certain amino acids, suggesting altered amino acid metabolism in the gut. However, the host utilization and metabolism of these amino acids remains unclear.

### Dynamic bacterial diversity

It has been reported that age-related changes in bacterial diversity are associated with different growing stages in commercial pigs [32, 33]. A longitudinal investigation of the gut microbiome in pigs from birth to market has been described in our previous study [21]. However, relatively few studies have tracked how sows’ rectal and vaginal bacterial diversity changed over parities. In the present study, bacterial alpha diversity exhibited different dynamic changes between the rectum and vagina. For the rectum, we observed a significant age-driven response with higher alpha diversity in every breeding and parturition stage than in the growth stage (9 weeks of age), consistent with previous research [22], suggesting that bacterial diversity of sows does not plateau until after sexual maturity (30 weeks of age). Comparing within parities, we found three of the four parities had higher alpha diversity on d 110 than on d 0 of gestation. However, the opposite was observed in the vagina, which tended to have a lower alpha diversity on d 110 of gestation. This finding on the vagina microbiome was supported by a previous study that late pregnant sows exhibited significantly lower alpha diversity when compared to their breeding stage [27]. Notably, this aspect was also previously reported in pregnant women, probably due to altered physiological conditions and sex hormone changes [34, 35]. Furthermore, in the current study, vaginal alpha diversity was associated strongly with parity, as it continuously declined as parity increased. Similar observations have been reported in women, with the richness and evenness of vaginal microbiota decreasing as gestational age increases [36]. However, the reason why microbial diversity decreases in the vagina but increases in the rectum with increasing parity is currently unexplained. Overall, these results revealed that age and pregnancy could both result in a change in bacterial diversity.

### Parity-related bacteria

Both parity and the microbiome have been identified as key factors in gestational health. A previous study has shown that the fecal microbiome during pregnancy exhibited significant differences between low and high parity in sows [37], and similar results have been reported in humans [38]. These studies all used samples from a single gut or vaginal source and were cross-sectional studies that ignored the potential impact of individual variation. Here, our data identified the distinguished bacteria from paired rectal and vaginal samples in sows that were repeatedly sampled throughout different parties. The rectum has been reported to contribute to the vaginal microbiota colonization [39]. Interestingly, we found that sows shared vaginal and rectal bacteria that were enriched at almost every stage, including *Prevotella*,* Veillonella*, *Streptococcus*, *Clostridium*, and *Lactobacillus*. Microbes exhibited stage-associated characteristics according to the growth and reproductive status of sows. Several ASVs, both enriched in the rectum and vagina at nine weeks, belong to the genus *Prevotella*, which has been reported to be related to feed efficiency and growth performance [40]. *Streptococcus* (ASV1) was enriched in parity 1 in both the vagina and rectum. It has been proven that rectal colonization with group B *Streptococcus* was the single strongest determinant of vaginal colonization [41]. Furthermore, group B *Streptococcus* exhibited a prevalence in the vagina and rectum of pregnant women [42]. *Lactobacillus* (ASV3), enriched in parity 2, has been shown to possess benefits in maintaining gut and vaginal health [43, 44]. We acknowledge that 16S rRNA sequencing technology cannot classify the ASVs at the species level. Metagenomic sequencing [45] will likely help us identify species and even bacterial strains, providing additional information for future investigations.

### Pregnancy-related bacteria

Profound physiological differences, such as hormone levels, have been reported between the pregnant and non-pregnant periods. The way in which bacteria in pregnancy are distinguished from non-pregnancy is of major interest to this study. Our data indicated an interesting phenomenon that both rectal and vaginal *Streptococcus* (ASV1) significantly decreased from the estrus to the late term of pregnancy, almost in each parity. This agrees with observations made in women [46, 47]. However, the effect of genus *Streptococcus* within different species on the body is contradictory. Some studies suggested that *Streptococcus pyogenes* and group B *Streptococcus* had adverse maternal outcomes [48], while other studies reported that *Streptococcus sanguinis* can exhibit an antimicrobial effect against exogenous pathogens [49]. Therefore, unclassified *Streptococcus* species, due to the sequence depth deficiency in our study, remain for further functional investigation. Another interesting point could be reflected in the decrease of *Anaerococcus* from estrus to late gestation in the vagina. *Anaerococcus* (ASV14) was enriched in the U4P group of the early breeding stage as the predictor of estrus and pregnancy. However, it decreased after pregnancy. *Anaerococcus* has also been identified in the vaginal microbes of sows with a low risk for pelvic organ prolapse [50]. Furthermore, *Anaerococcus* were enriched in the feces of weaned piglets with increased body weight performance [51]. Unlike pigs, *Anaerococcus* in humans seems to be more related to disease and is considered a pathogen [52, 53]. Therefore, we emphasize the need to better define the vaginal microbiome in pigs and its function associated with reproductive health in future studies. *Lactobacillus* is a key taxon in maintaining the vaginal ecosystem, providing colonization resistance for exogenous bacteria through lactate production [54]. Strikingly, four *Lactobacillus* species had higher relative abundance in pregnant women [46]. We also found that vaginal unclassified *Lactobacillales* (ASV433) were higher during the late term of pregnancy (d 110 of gestation) in each parity, revealing the crucial role of *Lactobacillus* in the vagina during the pregnancy period. Our findings revealed the specific bacteria that changed with pregnancy status in sows and may contribute to the investigation of how pregnancy-related microbes exhibit functional implications for the host.

### Shared bacteria between vagina and rectum

The gut and vaginal microbiota represent complex and diverse functions in the body. It has been indicated that several bacteria species may colonize both niches [55]. Interestingly, this study revealed that bacteria such as *Lactobacillus*, *Streptococcus*, *Prevotella 9*, and *Clostridium butyricum* were present in both the vagina and rectum at different time points. For example, *Lactobacillus* is responsible for fermenting carbohydrates and producing short-chain fatty acids in the gut, while maintaining a low pH to prevent the colonization of pathogens in the vagina. The dominant *Lactobacillus* species in the vagina is believed to originate from the gut [56], which is supported by studies indicating that oral probiotics can influence the composition of vaginal microbiota [57]. Similarly, the colonization of group B *Streptococcus* in the gut would increase the likelihood of its vaginal colonization, suggesting transfer from rectum to vagina [41]. These studies suggest a close relationship between the vaginal and gut microbiota. However, the precise mechanisms behind this connection remain unclear and require further investigation.

### Reproductive performance-associated bacteria

Various studies have shown that sows’ reproductive performance correlates with microbes. To predict sows with high reproductive efficiency in the nulliparous breeding stage, we documented and compared each sow’s accumulated total number of born alive piglets over the four parities. Lachnospiraceae family level in the rectum of sows has been reported to be negatively correlated with the number of piglets born alive and the birth weight of piglets [58]. Furthermore, Lachnospiraceae showed negative correlations with litter weight gain and average daily gain in sows [59]. This may be because Lachnospiraceae is associated with different intra- and extraintestinal diseases [60]. However, Lachnospiraceae has also been identified as a beneficial bacteria that produces short-chain fatty acids and degrades cellulose [61]. Consistent with previous studies, our data indicated that Lachnospiraceae were also listed as the reproductive performance-related bacteria in both the rectum and vagina, suggesting that carefully considering its controversial role in reproductive performance. Notably, we also found that *Prevotellaceae NK3B31 group* was listed as the reproductive-related predictors both in the rectum and vagina. Similarly, it has been reported that the *Prevotellaceae NK3B31 group* was enriched in sows with high-productive capacity at late pregnancy [62]. Meishan sows with increased litter sizes possessed a higher abundance of *Prevotellaceae UCG-001* in early pregnancy and *Prevotellaceae_UCG-003* during late pregnancy compared to Landrace × Yorkshire sows [63]. Our study also identified these two bacteria as reproductive performance-related predictors. *Streptococcus* was considered an important pathogen in pigs, leading to severe infections and diseases [64]. However, more recent research has reported that some species of *Streptococcus* have probiotic potential in pigs, regulating growth performance and gut health [65]. *Streptococcus* (ASV1), identified as the predominant bacteria related to reproductive performance, was also enriched in parity 1, revealing its potential role in promoting reproductive health in the early breeding stage.

### Limitations and improvements

To the best of our knowledge, this study is the first to comprehensively characterize changes in both rectal and vaginal microbiota in gilts, starting at 9 weeks of age and continuing through four parity cycles. Furthermore, we identified bacterial consortia that are significantly associated with reproductive performance and sow longevity. Despite the novelty of these findings, our study has a relatively small sample size. Future studies are warranted to validate these results in larger, more diverse populations and to improve the predictive accuracy of microbial composition on reproductive performance and longevity.

## Conclusions

In conclusion, this study investigates the rectal and vaginal bacteria of sows with increased reproductivity traits throughout four parities. The sows with longevity potential exhibited lower bacterial diversity and unique bacteria and potential function in the nulliparous breeding stage. We highlight and characterize the dynamics of the rectal and vagina microbiome of breeding sows in four parities. Parity-associated and pregnancy-related bacteria showed unique bacterial characteristics and composition, reflecting bacterial changes in response to different physiological periods. Sows with longevity potential showed better reproductive performance. Importantly, we observed the bacterial predictor for the total number of born-alive piglets in four parities. These findings contribute to the understanding of the parity-dependent dynamic microbiome for sows with longevity in reproduction and provide a research foundation for the role of microbes in regulating animal health and production.

## Supplementary Information

Additional file 1: Table S1. Diet composition for each stage of production (as-fed basis).

Additional file 2: Fig. S1. The bacterial diversity of sows with different breeding status. Fig. S2. The top bacterial genus of sows with different breeding status. Fig. S3. The bacterial correlation of predictors between U4P and non-U4P groups. Fig. S4. The top bacterial genus and ASV in the U4P group through four parities. Fig. S5. The reproductive performance between L4P and U4P. Fig. S6. The correlation between reproductive performance and bacterial ASVs.

## Data Availability

The data generated in this study were submitted to the National Center for Biotechnology Information (NCBI) Sequence Read Archive (SRA) database (www.ncbi.nlm.nih.gov/sra) and are available with BioProject accession number PRJNA 1137406 (available on December 1, 2024).

## Abbreviations

ANOSIM: Analysis of similarity
ASVs: Amplicon sequence variants
FDR: False discovery rate
KO: KEGG orthologous
LEfSe: Linear discriminant analysis effect size
PCoA: Principal coordinate analysis
