# Supplementary material for: The sow vaginal and gut microbiota associated with longevity and reproductive performance
Source: J Anim Sci Biotechnol. 2025 Jan 7;16:6. doi: 10.1186/s40104-024-01140-2 (PMC11705881; doi:10.1186/s40104-024-01140-2)
Supplement: Supplementary file 1 — Additional file 1: Table S1. Diet composition for each stage of production (as-fed basis). [file 40104_2024_1140_MOESM1_ESM.docx]

|  | Gilt development | | |  |  |
| --- | --- | --- | --- | --- | --- |
|  | Phase 1 | Phase 2 | Phase 3 | Gestation | Lactation |
| Ingredient, % |  |  |  |  |  |
| Corn, Yellow Dent | 69.40 | 77.35 | 77.35 | 74.82 | 60.17 |
| Soybean Meal 48 protein | 27.57 | 19.53 | 19.53 | 6.75 | 26.48 |
| Calcium carbonate | 1.08 | 1.25 | 1.25 | 1.23 | 1.27 |
| Monocalcium phosphate 21 | 0.58 | 0.50 | 0.50 | 1.04 | 0.71 |
| Sodium chloride | 0.54 | 0.55 | 0.55 | 0.68 | 0.53 |
| Biolys 77 Dry (60 Lys) | 0.39 | 0.40 | 0.40 | 0.38 | 0.34 |
| Vitamin Trace mineral premixes* | 0.25 | 0.25 | 0.25 | 0.25 | 0.25 |
| MHA dry | 0.10 | 0.07 | 0.07 | 0.07 | 0.05 |
| L-Thr | 0.09 | 0.09 | 0.09 | 0.15 | 0.10 |
| L-Trp | 0.00 | 0.01 | 0.01 | 0.02 | 0.00 |
| Wheat Middlings | 0.00 | 0.00 | 0.00 | 14.50 | 7.50 |
| Choline chloride dry 60 | 0.00 | 0.00 | 0.00 | 0.10 | 0.10 |
| Corn oil | 0.00 | 0.00 | 0.00 | 0.00 | 2.50 |
| Total: | 100.00 | 100.00 | 100.00 | 100.00 | 100.00 |
| Calculate |  |  |  |  |  |
| ME, kcal/kg | 3288 | 3293 | 3293 | 3219 | 3372 |
| NE, kcal/kg | 2448 | 2492 | 2492 | 2466 | 2522 |
| Protein, % | 18.6 | 15.4 | 15.4 | 11.5 | 18.6 |
| NDF, % | 8.6 | 8.7 | 8.7 | 12.4 | 10.3 |
| ADF, % | 3.5 | 3.3 | 3.3 | 3.4 | 3.6 |
| SID LYS, % | 1.09 | 0.9 | 0.9 | 0.62 | 1.05 |
| Analyzed Ca, % | 0.653 | 0.672 | 0.672 | 0.73 | 0.743 |
| Total P, % | 0.502 | 0.448 | 0.448 | 0.608 | 0.572 |
| STTD P, % | 0.386 | 0.35 | 0.35 | 0.486 | 0.44 |
| Analyzed Ca:Total P, Ratio | 1.301 | 1.5 | 1.5 | 1.201 | 1.299 |
| SID Lysine:NE kcal, Ratio | 4.274 | 3.509 | 3.509 | 2.488 | 4.007 |
| SID Leu:SID Lys, Ratio | 1.339 | 1.411 | 1.411 | 1.55 | 1.342 |
| SID Met+Cys:SID Lys, Ratio | 0.57 | 0.58 | 0.58 | 0.7 | 0.545 |
| SID Thr:SID Lys, Ratio | 0.62 | 0.63 | 0.63 | 0.76 | 0.64 |
| SID Trp:SID Lys, Ratio | 0.183 | 0.18 | 0.18 | 0.19 | 0.19 |
| SID Val:SID Lys, Ratio | 0.69 | 0.69 | 0.69 | 0.726 | 0.71 |
| SID Iso:SID Lys, Ratio | 0.628 | 0.611 | 0.611 | 0.581 | 0.641 |
| SID His:SID Lye, Ratio | 0.418 | 0.421 | 0.421 | 0.452 | 0.431 |

Supplemental Table 1, Diet composition for each stage of production (as-fed basis).

Gilts were fed three-phase regimes during the development periods: phase 1 (22.7 to 56.8 kg), Phase 2 (56.8 to 90.8 kg), and Phase 3 (90.8 to 131.7 kg). A gestation diet was offered from 26 weeks of age throughout the entire gestation period until they were placed in a lactation facility on day 110 of gestation where a lactation diet was fed.

*provided 5511.5 IU Vitamin A, 500 IU Vitamin D, 0.05 mg 25(OH)D3, 66.14 IU Vitamin E, 27.6 mg Niacin, 8.3 mg Riboflavin, 1.1 mg Pyridoxin, 0.03 mg Vitamin B12, 27.56 mg Pantothenic acid, 2.2 mg Folic acid, .22 mg Biotin, 0.3 mg Selenium, 0.3 mg Iodine, 16.53 mg Copper, 110.1 mg Iron, 33.07 mg Manganese, and 110.12 mg Zinc /kg of complete feed.
