# Supplementary material for: The sow vaginal and gut microbiota associated with longevity and reproductive performance
Source: J Anim Sci Biotechnol. 2025 Jan 7;16:6. doi: 10.1186/s40104-024-01140-2 (PMC11705881; doi:10.1186/s40104-024-01140-2)
Supplement: Supplementary file 2 — Additional file 2: Fig. S1. The bacterial diversity of sows with different breeding status. Fig. S2. The top bacterial genus of sows with different breeding status. Fig. S3. The bacterial correlation of predictors between U4P and non-U4P groups. Fig. S4. The top bacterial genus and ASV in the U4P group through four parities. Fig. S5. The reproductive performance between L4P and U4P. Fig. S6. The correlation between reproductive performance and bacterial ASVs. [file 40104_2024_1140_MOESM2_ESM.docx]

**
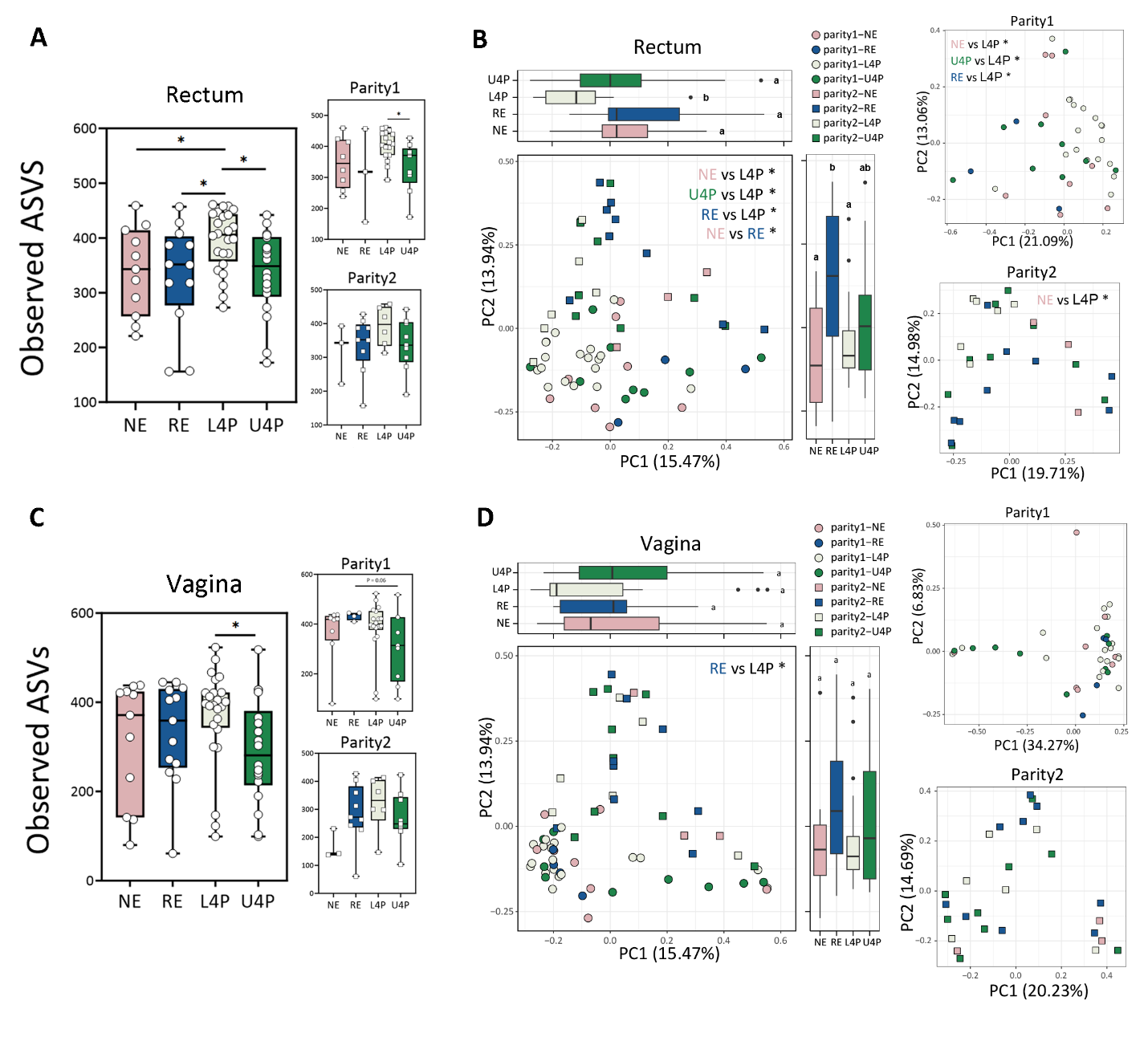
**

**Figure S1 The bacterial diversity of sows with different breeding status**

Boxplot illustrating observed ASVs between NE, RE, L4P, and U4P groups in the first 2 parities in the rectum (A) and vagina (C). Principal coordinate analysis (PCoA) plots illustrating the bacterial composition between NE, RE, L4P and U4P groups in the first 2 parities based on the Bray-Curtis distances in the rectum (B) and vagina (D). Asterisks mean statistically significant difference: * *P* value < 0.05.

**
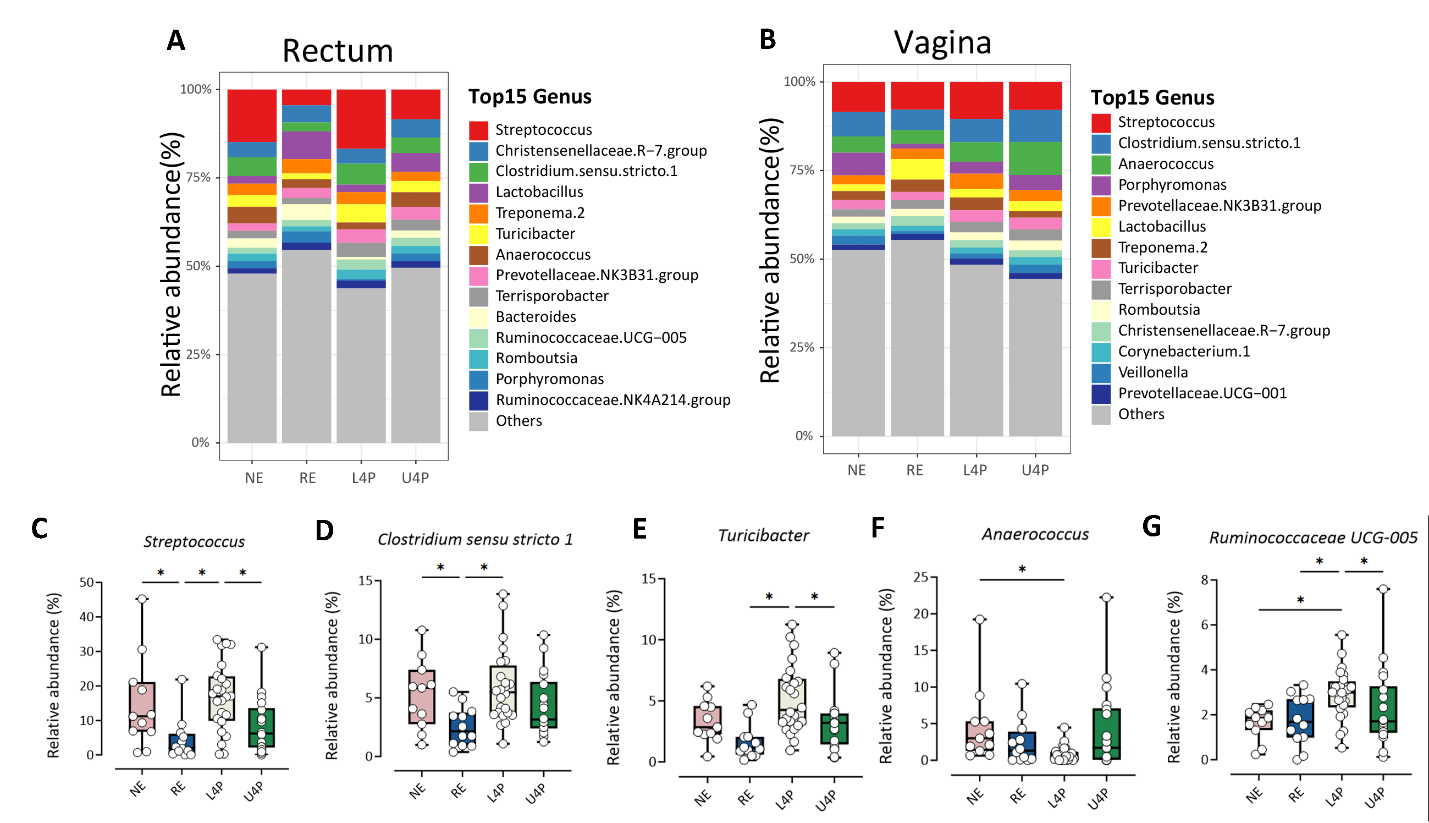
**

**Figure S2 The top bacterial genus of sows with different breeding status**

Bar chart showing the relative abundance of top 15 bacterial genera between NE, RE, L4P, and U4P groups in the first 2 parities in rectum (A) and vagina (B). Boxplot illustrating the relative abundance of *Streptococcus* (C), *Clostridium sensu stricto 1* (D), *Turicibacter* (E), *Anaerococcus* (F), *Ruminococcaceae UCG-005* (G) between NE, RE, L4P and U4P groups in the first 2 parities in the rectum. Asterisks mean statistically significant difference: * *P* value < 0.05.

**
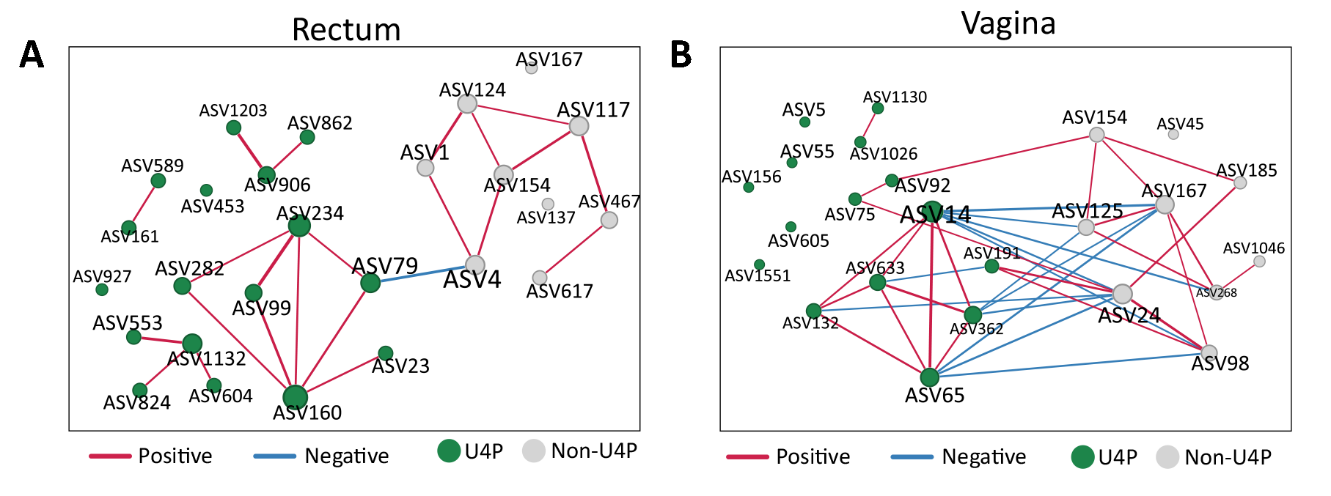
**

**Figure S3 The bacterial correlation of predictors between U4P and non-U4P groups.**

The network of distinguished ASVs correlation between U4P and non-U4P group in the rectum (A) and vagina (B). The correlations between ASV were shown based on the Spearman rho over 0.4 or less than -0.4 and adjusted *P* value < 0.05.


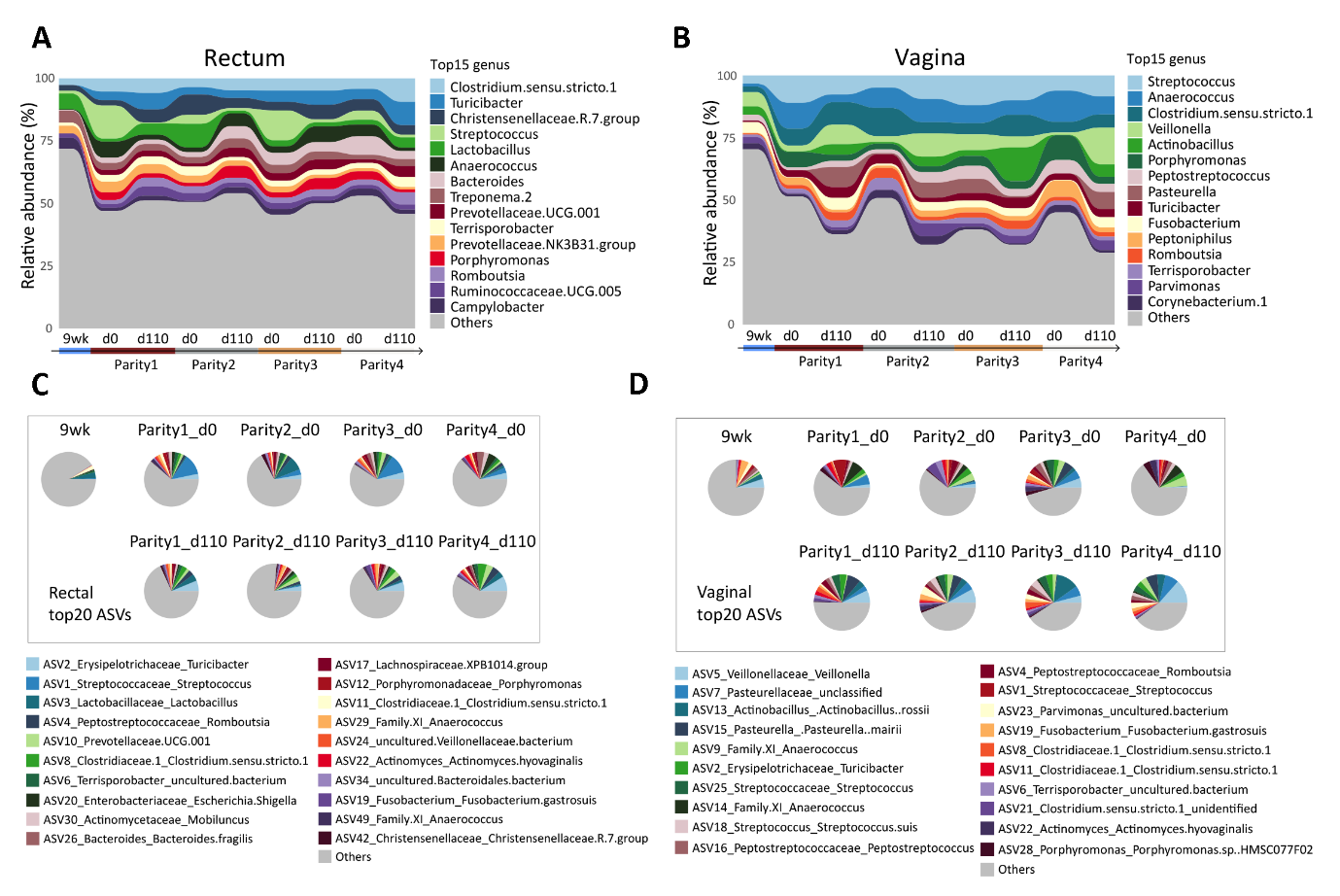


**Figure S4 The top bacterial genus and ASV in the U4P group through four parities.**

Bar chart showing the relative abundance of top 15 bacterial genera through four parities in rectum (A) and vagina (B). Pie chart showing the relative abundance of top 20 bacterial ASV through four parities in rectum (C) and vagina (D).


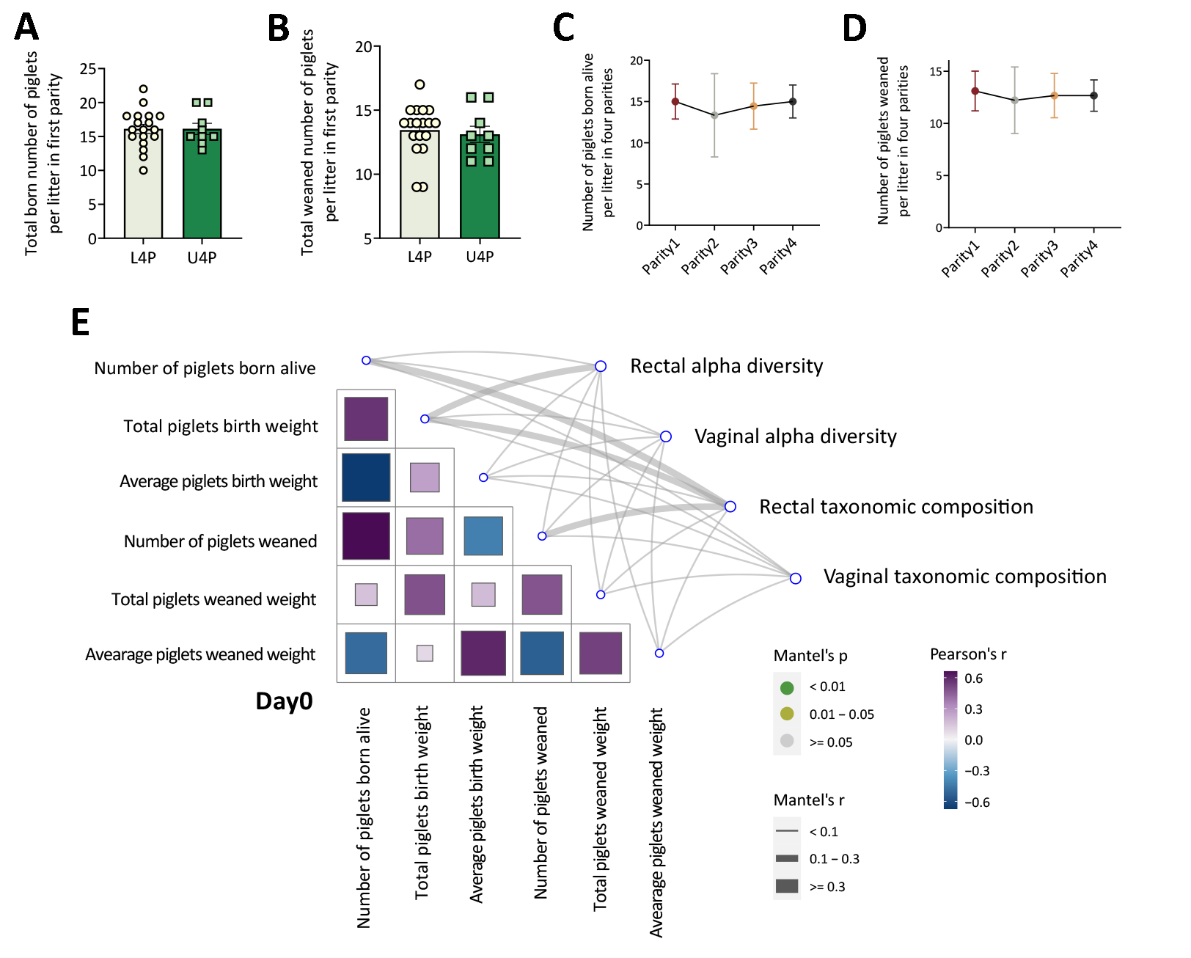


**Figure S5 The reproductive performance between L4P and U4P**

Bar chart illustrating total birth number of piglets (A), total weaned number of piglets (B) per litter between L4P and U4P in the first parity. Line chart illustrating total birth number of piglets (C), total weaned number of piglets (D) per litter of U4P groups through four parities. The one-way ANOVA was used to detect significant differences for multiple comparisons with Fisher’s LSD test. The Mantel t-test showing the correlation between reproductive performance and bacterial diversity and composition day0 (I). The line between the two points represents the relationship between reproductive performance and bacterial characteristics, the thickness represents the size of Mantel’s r, and the color represents the range of Mantel’s p values.


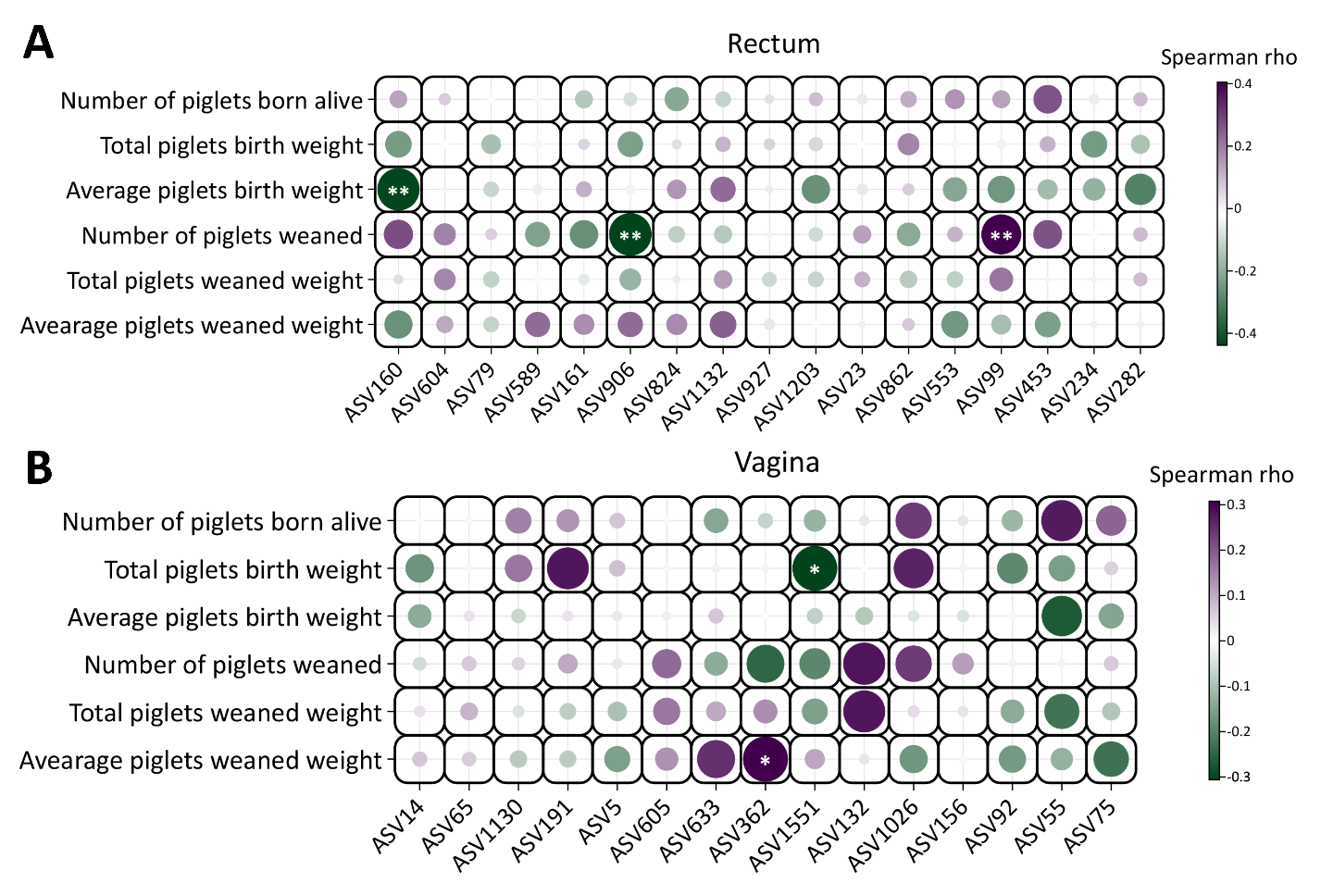


**Figure S6 The correlation between reproductive performance and bacterial ASVs**

Correlation between reproductive performance and predicted ASVs for U4P groups, respectively in the rectum (A) and vagina (B). The purple represents a significant positive correlation, the green represents a significant negative correlation, and the darker color represents stronger correlations, which are based on Spearman’s correlation. Asterisks mean statistically significant difference: * *P* value < 0.05.
